# The taxonomic identities of *Pholidota wenshanica* and *P. subcalceata* (Orchidaceae, Coelogyninae)

**DOI:** 10.3897/phytokeys.136.46705

**Published:** 2019-12-19

**Authors:** Lin Li, Min Qin, Wan-Yao Wang, Song-Jun Zeng, Guo-Qiang Zhang, Zhong-Jian Liu

**Affiliations:** 1 Key Laboratory of Plant Resources Conservation and Sustainable Utilization, South China Botanical Garden, Chinese Academy of Sciences, Guangzhou 510650, China South China Botanical Garden, Chinese Academy of Sciences Guangzhou China; 2 University of Chinese Academy of Sciences, Beijing 100049, China University of Chinese Academy of Sciences Beijing China; 3 Hangzhou Heyi Gene Technology Co. Ltd., Hangzhou 310000, China Hangzhou Heyi Gene Technology Co. Ltd. Hangzhou China; 4 Key Laboratory of National Forestry and Grassland Administration for Orchid Conservation and Utilization, The National Orchid Conservation Center of China and The Orchid Conservation and Research Center of Shenzhen, Shenzhen, 518114, China Fujian Agriculture and Forestry University Fuzhou China; 5 Key Laboratory of National Forestry and Grassland Administration for Orchid Conservation and Utilization at College of Landscape Architecture, Fujian Agriculture and Forestry University, Fuzhou, 350001, China The National Orchid Conservation Center of China Shenzhen China

**Keywords:** China, emended description, orchid taxonomy, Vietnam

## Abstract

*P. wenshanica* S.C.Chen & Z.H.Tsi and *P. subcalceata* Gagnep. have long been recognized as synonyms of *P. leveilleana* Schltr. In the present study, detailed morphological comparisons suggest that specimens referred to as *P. wenshanica* and *P. subcalceata* differ significantly in both vegetative and floral characters from those of *P. leveilleana*. Here we resurrect *P. wenshanica* and *P. subcalceata* as independent species. Key diagnostic characters essential for delineating identities of these species are presented.

## Introduction

The orchid genus *Pholidota* Lindl. ex Hook. was established by Hooker (1825: pl. 138). The generic epithet is from the Greek *pholidotos*, referring to the imbricate bracts of the inflorescence of some species (Pridgeon et al. 2005). As currently circumscribed, the genus is classified within the subtribe Coelogyninae, subfamily Epidendroideae, comprising about 30 species, distributed from Pakistan, India, Sri Lanka, Nepal, Bhutan, Myanmar, S China, Taiwan, Indo-China and the Malesian region into the SW Pacific (Seidenfaden 1986; de Vogel 1988; Seidenfaden and Wood 1992; Pearce and Cribb 2002; Pridgeon et al. 2005).

The taxonomic status of the Chinese species *P. wenshanica* S.C.Chen & Z.H.Tsi (1988: 7) has long been in doubt. Soon after its publication in the same year, this species has been placed in synonym with *P. leveilleana* Schltr. (1913: 107) by de Vogel (1988) while revising the genus *Pholidota*. There have been no further references to that species, except in the protologue (Chen and Tsi 1988) and subsequent reports (Chen and Tsi 1998; Chen 1999). In Flora Reipulicae Popularis Sinicae, Chen (1999) regarded this species as an independent species and consistently named *P. wenshanica*. However, in the revised edition Flora of China, Chen and Wood (2009) followed the treatment of de Vogel (1988) and reduced it as the synonym of *P. leveilleana* with some doubts. According to the addendum made by de Vogel (1988): “ I have not seen the holotype, Tsi 223 (PE), but the description and the line drawings do agree so very well with *P. leveilleana* Schltr. that I am convinced that it is conspecific with that species”. These more or less ambiguous treatments of *P. wenshanica* caught our attention.

Comparison of *Pholidota* specimens collected from different localities in China showed those representing *P. wenshanica* could be distinguished from *P. leveilleana* on the basis of several morphological characters recognized in this study. Further investigation revealed that a Vietnamese species *P. subcalceata*, which has been treated as a synonym of *P. leveilleana*, also differs in its unique characters. The present paper includes confirmation of the validities of *P. wenshanica* and *P. subcalceata*, assessments of diagnostic characters of these species and descriptions of newly recognized diagnostic characters.

## Material and methods

To clarify the taxonomic status of *Pholidota wenshanica* in China, morphological studies were performed using specimens deposited at herbaria E, GXMG, IBK, IBSC, KUN, P and PE, and online databases such as JSTOR Global Plants (http://plants.jstor.org) and Chinese Virtual Herbarium (http://www.cvh.ac.cn/), with special focus on the type specimens. The only type of *P. wenshanica* was thoroughly examined and compared with various specimens of *P. leveilleana* from China including some type materials of its synonym *P. subcalceata* from Vietnam. Relevant literature, including the protologue, was consulted. We also conducted field investigations in the type localities of *P. wenshanica* and *P. leveilleana*. Living plants were collected and transplanted to the nursery of South China Botanical Garden (**GDGM**) for further observation. Measurements and photographs of the fresh material were made under a stereomicroscope Olympus MD-90. Herbarium abbreviations follow Index Herbariorum (Thiers 2015, http://sweetgum.nybg.org/science/ih/).

## Results and discussion

*Pholidota wenshanica* S.C.Chen & Z.H.Tsi was described based on a specimen collected from Wenshan County, Yunnan (Chen and Tsi 1988). According to the protologue, this species is characterized by having fusiform-cylindrical pseudobulbs with two apical leaves, well-spaced on creeping rhizomes, lanceolate-oblong leaves.

**Figure 1.**
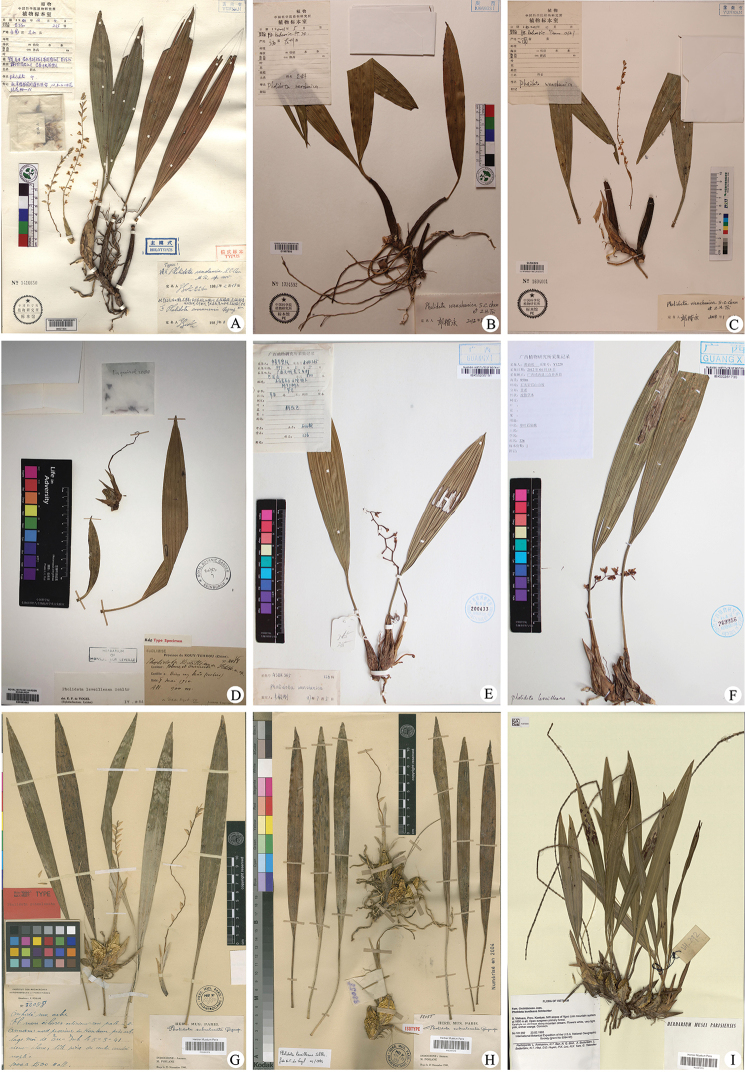
The types and selected specimens of *P. wenshanica* (**A–C**), *Pholidota leveilleana* (**D–F**) and *P. subcalceata* (**G–I**) **A** holotype sheet of *P. wenshanica***B** China, Guangxi, Longzhou, *HK Kadoorie PT 714* (PE) **C** China, Yunnan, *HK Kadoorie Team 2361* (PE) **D** holotype sheet of *P. leveilleana***E** China, Guangxi, *China-UK Expedition Team ASBK365* (IBK) **F** China, Guangxi, *Y.S. Huang Y1229* (IBK) **G** Holotype sheet of *P. subcalceata***H** isotype sheet of *P. subcalceata***I** Vietnam, Kontum, *L.V. Averyanov & al.*, *VH 292* (P).

*Pholidota leveilleana* Schltr. was described by Schlechter (1913) on the basis of a specimen collected by J. Esquirol from Guizhou (= Kweichow or Kouy-tchou), China. In the protologue, the author stated that the species is easily distinguished from the related species *P. yunnanensis*Rolfe (1903: 24) by unifoliate pseudobulbs (vs. two-leaved), loose (vs. tight) inflorescences with larger, white and crimson-red (vs. white) flowers. Unfortunately, the type sheet of *P. leveilleana* is nearly complete (Fig. 1D). In his monograph, de Vogel (1988) pointed out that the type specimen of *P. leveilleana* has only one leaf because it is a weakly developed plant, and thus this character cannot be used to distinguish these species. At the same time, he placed *P. subcalceata* Gagnep. (1950: 508) as the synonym of *P. leveilleana*. *P. subcalceata* was described based on two collections from Vietnam (Fig. 1G, H). Gagnepain (1950) stated in the protologue that *P. subcalceata* has two linear to lanceolate leaves ca. 30 cm long. De Vogel’s treatment has long been accepted until Chen (1999) noticed the clear difference in the leaf number of *P. leveilleana*. He placed *P. leveilleana* in section Pholidota, which is characterized by pseudobulbs with only one leaf.

Based on our close examination of the type specimens of *P. wenshanica* (Fig. 1A), *P. leveilleana* (Fig. 1D) and *P. subcalceata* (Fig. 1G, H), and of other specimens so named in E, P and PE (Fig. 1), as well as the field observation, we are convinced that the leaf number is unlikely to change with growth in either *P. leveilleana* or *P. wenshanica*. Although the type specimen of *P. leveilleana* is not seemingly perfect, the morphology of specimen J. Esquirol no. 2088 (Fig. 1D) actually conforms most closely to the diagnosis given in Schlechter (1913).

**Figure 2.**
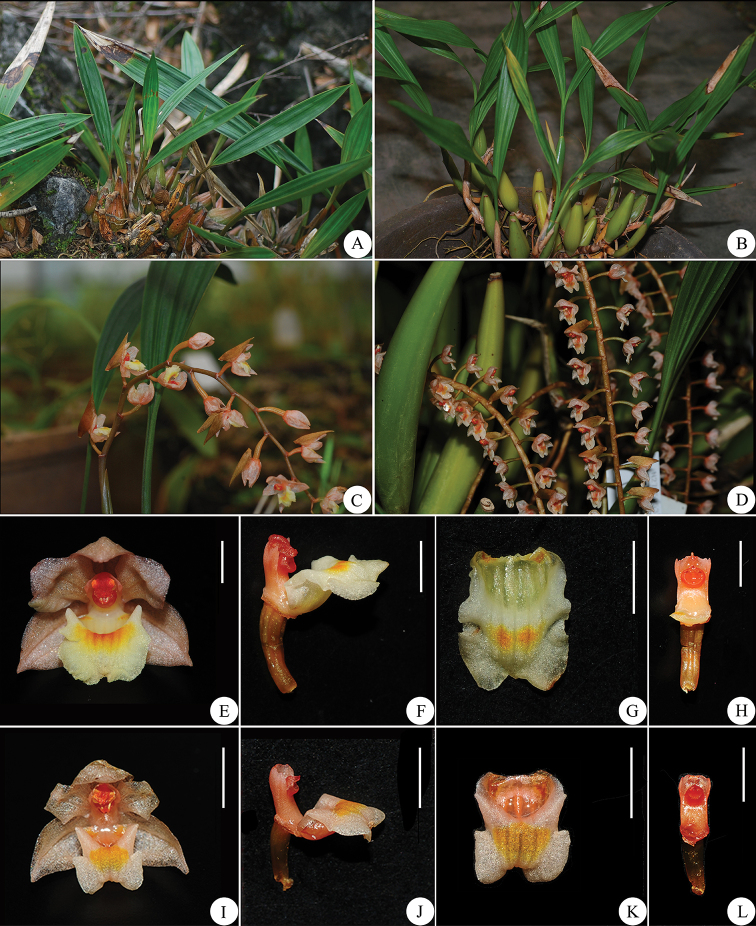
The morphology of *Pholidota leveilleana* (**A, C, E–H**) and *P. wenshanica* (**B, D, I–L**) **A–B** habit **C–D** inflorescences **E, I** flower, frontal view **F, J** column and lip, lateral view **G, K** lip, ventral view **H,L** column, front view. Scale bars: 5 mm (**E, I**), 3 mm (**F–H, J–L**).

Morphological examinations indicate significant differences among these species. *P. wenshanica* is easily distinguished from the other two species by fusiform-cylindrical pseudobulbs, much more slender (7−8 cm long) and well apart (2 cm intervals or distance), with two oblong-lanceolate leaves, up to 30 cm long and ca. 3.5 cm wide. In floral morphology, *P. wenshanica* can be readily distinguished from *P. leveilleana* by the flower number and size, as well as the details of flowers. The former has distinctly more (30–40) flowers arranged alternately on the almost straight rachis, whereas *P. leveilleana* has fewer (12–18) flowers on the weakly zig-zag rachis (Fig. 2). On the other hand, the pseudobulbs of *P. subcalceata* are ovoid and close together, which are superficially similar to those of *P. leveilleana*, but carry two apical linear leaves. The size of its leaves varies from 15−30 cm in length and ca. 2.5 cm in width, whereas the leaves of *P. leveilleana* ca. 3.5 cm in width. In addition, *P. subcalceata* has synanthous inflorescence, with partially developed leaves at anthesis. A comprehensive morphological comparison among these species is presented in Table 1.

**Table 1.** Morphological comparison of *Pholidota wenshanica*, *P. leveilleana* and *P. subcalceata*.

**Characters**	***P. wenshanica***	***P. leveilleana***	***P. subcalceata***
Pseudobulbs	fusiform-cylindrical, 7–8 cm × 6–8 mm, 1.5–2 cm apart, two-leaved	ovoid to conical-ovoid, 2.5–4.5 × ca. 3.5 cm, almost densely placed, unifoliate	narrowly ovoid to broadly fusiform, 1.5–3.5 × ca. 2 cm, densely placed, two-leaved
Leaves	lanceolate-oblong 25–30 × 3–3.5 cm	narrowly elliptic to elliptic-lanceolate 15–25 × 2–3.5 cm	linear to linear-lanceolate 15–30 × 1–2.5 cm
Petiole	3.5–4 cm long	4.5–7 cm long	4–8 cm long
Inflorescence	peduncle 3.5–4 cm, raceme with 30–40 flowers; rachis almost straight	peduncle ca. 7 cm, raceme with 12–18 flowers; rachis weakly zig-zag	peduncle 3–4 cm, raceme with 18–25 flowers; rachis almost straight
Lip	subovate when flattened, lateral lobes inconspicuous	broadly oblong when flattened, lateral lobes inconspicuous	subpandurate when flattened, trilobed, lateral lobes prominent
Hypochile	deeply saccated, with 4 prominent fleshy keels or carinae	shallowly cupular, with 3 thickened veins	deeply saccated, with 3 thickened veins
Epichile	transversely elliptic, apex deeply notched into 2 broadly rounded lobes	transversely oblong, apex shallowly emarginate	reniform-orbicular, apex emarginate and truncate-subbilobed
Column	2.5–3 mm, apex narrowly winged	3.5–4 mm, apex broadly winged	3 mm, apex broadly winged
Stelidia	rounded, with obtuse teeth along the upper margin	sharp, with conspicuous acute teeth along the upper margin	short and rounded, with obtuse teeth along the upper margin

An additional specimen’s survey indicates that several collections previously identified as *P. wenshanica*, such as *China-UK Expedition Team ASBK365* (IBK) (Fig. 1E) actually belong to *P. leveilleana*, with their unifoliate pseudobulbs closely placed.

In distribution, both *P. wenshanica* and *P. leveilleana* are endemic to southwestern China. *P. leveilleana* occur in W to N Guangxi and S Guizhou. *P. wenshanica* is currently only found in SE Yunnan and SW Guangxi. *P. subcalceata* is endemic to southern Vietnam. The more or less disjunct distributions clearly indicate that *P. wenshanica* and *P. subcalceata* should be considered as distinct species.

### Taxonomic treatment

#### Pholidota wenshanica

Taxon classificationPlantaeAsparagalesOrchidaceae

S.C.Chen & Z.H.Tsi

FBD1ED77-892E-5958-B580-44A2221DBD88

Figs 1 , 2

Pholidota wenshanica S.C. Chen & Z.H. Tsi, Bull. Bot. Res. 8(1): 7, fig. 1. 1988. Type: – CHINA. Yunnan, Wenshan County, cult. in Hort. Bot. Beijing, 4 Dec. 1984, *Z.H. Tsi 223* (holotype PE!).

##### Emended description.

Plants lithophytic, up to 35 cm high; rhizome creeping, terete, 8–10 mm in diam., enclosed by coriaceous scales. Pseudobulbs fusiform-cylindrical, 7–8 × 6–8 mm, 2 cm apart, tapering to based and top, smooth or longitudinally wrinkled when dried, base usually enclosed by coriaceous sheaths. Leaves 2 per pseudobulb, arising from pseudobulb apex, oblong-lanceolate, 25–30 × 3–3.5 cm, apex acuminate, base cuneate, lamina glossy green, more or less coriaceous; petioles 3–4.5 cm long. Inflorescence a racemose, proteranthous, glabrous, pendulous, 17–19 cm long; peduncle 3.5–4 cm long, very thin, covered by sterile bracts at base of rachis; rachis slender, 13.5–20 cm long, almost straight or weakly zig-zag, laxly 30–40-flowered; floral bracts broadly rhombic-ovate, papyraceous, 4 × 6 mm, folded along the midrib, caduceus at anthesis. Flowers pinkish white, ca. 5 mm in diam., lip salmon-pink, tinged with yellowish-brown or orangish-brown blotches. Pedicel and ovary 3–4 mm long. Sepals subequal; dorsal sepal elliptic, 5 × 3 mm, apex acute, obscurely 7-nerved; lateral sepals ovate, slightly oblique, 5–6 mm long, strongly keeled on the back, apex shortly acuminate, obscurely 7-nerved. Petals ovate, 4 × 3mm, apex obtuse, obscurely 3–5-nerved; Labellum subovate in outline, 4–5 long; hypochile deeply saccated, with 4 prominent fleshy keels or carinae; epichile transversely elliptic, 4–5 mm wide, margin inconspicuously undulate, apex deeply notched into 2 broadly rounded lobes. Column stout, 2.5–3 mm long; apex narrowly winged, foot absent; stelidia obtuse, up margin with an inconspicuous rounded wing near the apex; anther incumbent, top retuse to rounded; pollinia 4 in 2 pairs, connected by caudicles to a sticky substance, pyriform, ca 0.5 by 0.4 mm; stigma broadly ovate; rostellum large, broadly triangular. Flowering in late November and early December. Capsule not seen.

##### Distribution and habitat.

*Pholidota wenshanica* is currently known only from SE Yunnan (Wenshan) and SW Guangxi (Longzhou), China, where it grows as epiphyte on tree trunks or as lithophyte on somewhat shady slopes or on the edge of forests, often in exposed places, with elevations ranging from 1200 m to 1500 m a.s.l.

##### Additional specimens examined.

China. Yunnan, Malipo County, Tiechang Town, at 1500 m alt., 13 Dec. 1992, *Tsi s.n.* (PE). China. Yunnan, *precise* locality unknown, Nov. 2001, *HK Kadoorie Team 2361* (PE). China. Guangxi Zhuang Autonomous Region, Longzhou County, May 2001, *HK Kadoorie PT 714* (PE).

#### Pholidota leveilleana

Taxon classificationPlantaeAsparagalesOrchidaceae

Schlechter

DC4C84A0-054D-501F-88FD-BAB7FF72E1F4

Figs 1 , 2

Pholidota leveilleana Schlechter, Repert. Spec. Nov. Regni Veg. 12: 107. 1913. Type: – CHINA. Guizhou (Kouy-tcheou), Huishui County, Tian sheng qiao (Tien-sey-kao), at alt. 900m, 8 May 1910, *J. Esquirol 2088* (holotype E!).

##### Emended description.

Lithophytic or occasionally epiphytic plants with short and stout rhizomes, up to 30 cm high. Pseudobulbs borne close together, ovoid or conical-ovoid, often longitudinally sulcate, 2.5–4.5 cm × 8–12 mm, ca. 3.5 cm in diam., basally usually enveloped by scarious sheaths. Leaf solitary, arising from pseudobulb apex, narrowly elliptic or narrowly elliptic-lanceolate, 15–25 × 2–3.5 cm, papery, plicate, leaf vernation prominent, base contracted into a distinct petiole, apex short acuminate, lamina dark green to bluish green; petiole 4.5–8 cm. Inflorescence arising from base of mature pseudobulbs, often pendulous, 13–18 cm or longer; peduncle 4.5–7 cm long; rachis weakly zig-zag, laxly 12–18-flowered; floral bracts deciduous, elliptic or broadly ovate, papyraceous, 7 × 9 mm. Flowers pinkish white or salmon-pink, ca. 5 mm in diam., lip white or greenish white, tinged with orangish yellow or carmine red blotches, anther and stigma red; pedicel and ovary 3–4 mm. Sepals broadly ovate-elliptic, 5–7 × 4–6 mm, 7-veined, acute; lateral sepals dorsally carinate. Petals ovate-elliptic, 4–5 × 2.5–3 mm, 3–5-veined, obtuse; lip broadly oblong in outline, 5–6 × 3 mm, contracted into epichile and hypochile at apical 2/3; hypochile shallowly cupular in center, margin spreading horizontally, with 3 thickened veins extending from base to above middle; epichile transversely oblong or elliptic, 4–5 mm wide, apex emarginate, slightly undulate margined. Column 3.5–4 mm, apex broadly winged; stelidia sharp, with conspicuous acute teeth along upper margin; anther broadly elliptic in outline, top truncate to retuse; pollinia 4 in 2 pairs, connected by caudicles to a sticky substance, pyriform, ca 0.5 by 0.4 mm; stigma suborbicular; rostellum semi-orbicular. Flowering in April and May. Capsule narrowly obovoid, ca. 2 cm × 5–6 mm; fruiting pedicel 2–3 mm. 1.2–1.5 cm in diam.

##### Distribution and habitat.

*Pholidota leveilleana* is endemic to N and W Guangxi (Luocheng, Du’an, Jingxi, Nandan, Huanjiang, Tian’e, Fengshan, Napo), S Guizhou (Huishui), China, where it grows as lithophyte in sparse forests and shaded rocks, with elevations ranging from 500 m to 900 m a.s.l.

##### Additional specimens examined.

China, Guangxi Zhuang Autonomous Region, Hechi City, Luocheng Molao Autonomous County, Xunle Miao Ethnic Township, 11 Mar. 2013, *Luocheng County Exped. 451225130311036LY* (GXMG, *IBK*); Luocheng Mulao Autonomous County, 23 Jun. 1939, *W. Chen 84075* (PE). Guangxi Zhuang Autonomous Region, Hechi City, Huanjiang Maonan Autonomous County, Xunle Miao Ethnic Township, 26 Apr. 2013, *Huanjiang County Exped. 451226130426003LY* (GXMG, IBK); Huanjiang Maonan Autonomous County, Mulun Natural Reserve, 25 Apr. 2008, *W.B. Xu & Y. Liu 08025* (IBK); Mulun Natural Reserve, 25°06'43"N, 108°00'13"E, 27 Dec. 2008, *W.B. Xu*, *Y.Y. Liang*, *Y.S. Huang & X.X. Ye*, *Liuyan 0156* (KUN); Huanjiang Maonan Autonomous County, Mulun Town, NE Zhonglun, 10 Aug. 1994, *Mulun Exped. M0117* (PE). Guangxi Zhuang Autonomous Region, Tian’e County, 5 May 1997, *China-UK Expedition Team ASBK365* (IBK); Tian’e County, limestone Mt. at the junction of Lingdang and Liupai Town, 10 Aug. 1958, *Z.T. Li 601198* (PE). Guangxi Zhuang Autonomous Region, Du’an Yao autonomous County, Shangfu Township, *Y.K. Li P01539* (IBSC, PE). Guangxi Zhuang Autonomous Region, Fengshan County, Jinya Town, 24°38'4326.50"N, 106°45'00.96"E, 29 Mar. 2013, *H.Z. Lv*, *L.H. Liu & H.F. Chen 451223130329080LY* (GXMG). Guangxi Zhuang Autonomous Region, Nandan County, Lihu Town, 26 Jun 1937, C. Wang 40914 (IBSC, PE). Guangxi Zhuang Autonomous Region, Jingxi County, Sanhe Town, 15 Apr. 2012, *Y.S. Huang Y1229* (IBK); Jingxi County, Renzhuang Town, 14 Sep. 2006, *Y. Liu & W.B. Xu 0153* (IBK); Jingxi County, Longbang Town, Damo Village, 23 Apr. 2011, *F.Y. Huang & Z.H. LV LHZJX0248* (GXMG). Guangxi Zhuang Autonomous Region, Napo County, Chengxiang Town, 12 Apr. 1998, *H.N. Qin & al*. *506* (IBSC, PE). Guangxi Zhuang Autonomous Region, Luchen (Luocheng?), 27 May 1928, *anonymous 5405* (IBSC; PE); Guangxi Zhuang Autonomous Region, precise locality unknown, 25 Aug. 1935, *S.P. Ko 55619* (PE). CHINA. Guizhou (Kouy-tchou), Dushan County, 13 Jul. 1959, *Lipo Expe.1072* (PE).

#### Pholidota subcalceata

Taxon classificationPlantaeAsparagalesOrchidaceae

Gagnepain

AE41F896-85CF-538A-ADA7-1CFCBAD8F1DB

Pholidota subcalceata Gagnepain, Bull. Mus. Natl. Hist. Nat., sér. 2, 22: 508, 1950.

##### Type.

Vietnam. Annam, North Kon Tum, near Moi village, at 1000–1500 m alt., 25 Nov. 1941, M. Poilane *32058* (holotype P! Isotype P!). Fig. 1. Emended description Epiphytic or occasionally terrestrial plants with short and stout rhizomes, up to 1 m high. Pseudobulbs borne close together, narrowly ovoid to broadly fusiform, often longitudinally sulcate, 1.5–3.5 cm × 8–15 mm, ca. 2 cm in diam., basally usually enveloped by scarious sheaths. Leaves 2 per pseudobulb, arising from pseudobulb apex, linear to linear-lanceolate, 15–30 × 1–2.5 cm, somewhat coriaceous, base contracted into a distinct petiole, apex short acuminate, lamina dark green to bluish green; petiole 4–8 cm. Inflorescence arising from rather young pseudobulbs with just developing very young leaves, synanthous, often pendulous, ca. 23 cm long; peduncle 4–8 cm long; rachis almost straight or weakly zig-zag, laxly 18–25-flowered; floral bracts deciduous, broadly ovate-rhombic, 9 ×10 mm, paperaceous. Flowers white or slightly tinged with pink, ca. 5 mm in diam., lip white, tinged with pinkish yellow or yellow blotches, anther pink; stigma red; pedicel and ovary 3–4 mm. Sepals broadly ovate-elliptic, 5–7 × 4–6 mm, 7-veined, acute; lateral sepals dorsally carinate. Petals ovate-elliptic, 4–5 × 2.5–3 mm, 3–5-veined, obtuse; lip subpandurate when flattened, 5 × 3–4 mm, trilobed, with prominent lateral lobes; hypochile deeply saccated, with 3 thickened veins; epichile reniform-obicular, 4–5 mm wide, apex emarginate and truncate-subbilobed, slightly undulate margined. Column 3 mm, apex broadly winged; stelidia short and rounded, with obtuse teeth along the upper margin; anther broadly elliptic in outline, top convex and rounded; pollinia 4 in 2 pairs; stigma transversally reniform. Flowering in March and April. Capsule not seen.

##### Distribution and habitat.

*Pholidota subcalceata* is endemic to the Central Highlands of southern Vietnam, north to Kon Tum, south to Lam Dong, where it grows as epiphyte on old trees in montane broadleaved forest in open areas and along streams, and occasionally grows as terrestrial herb, with elevations ranging from 1000–1800 m a.s.l.

##### Additional specimens examined.

Vietnam. Prov. Lam Dong, distr. Lac Duong, municipalite Da Chay, 35 km to NE from Dalat city, 12°08'N, 108°39'E, at 1450 m alt., 19 Mar. 1997, *L.V. Averyanov*, *N.Q. Binh & P.K. Loc*, *VH 2897* (P). Vietnam. Prov. Lam Dong, distr. Lac Duong, municipalite Da Chay, 35 km to NE from Dalat city, 12°09'N, 108°41'E, at 1700–1800 m alt., 7 Apri. 1997, *L.V. Averyanov*, *N.Q. Binh & P.K. Loc*, *VH 3753* (P). Vietnam. Prov. Lam Dong, distr. Lac Duong, municipalite Da Chay, 26–28 km to NE from Dalat city, 12°07'N, 108°36'E, at 1500–1700 m alt., 4 Oct. 1997, *L.V. Averyanov*, *N.Q. Binh & P.K. Loc*, *VH 3842* (P). Vietnam. Prov. Kon Tum, NW slopes of Ngoc Linh mountain system, at 1600 m alt., 23 Feb. 1995, *L.V. Averyanov & al.*, *VH 290* (P); *L.V. Averyanov & al.*, *VH 291* (P); *L.V. Averyanov & al.*, *VH 292* (P); *L.V. Averyanov & al.*, *VH 294* (P);

## Supplementary Material

XML Treatment for Pholidota wenshanica

XML Treatment for Pholidota leveilleana

XML Treatment for Pholidota subcalceata

## References

[B1] Chen SC (1999) *Pholidota* Lindl. ex Hook. In: Chen SC Tsi ZH Lang KY Zhu GH (Eds) Flora Reipublicae Popularis Sinicae (Vol.18). Science Press, Beijing, 386–400. [in Chinese]

[B2] Chen SC Tsi ZH (1988) Duae species novae Orchidacearum sinicarum. Bulletin of Botanical Research 8(1): 7–8 [fig. 1]

[B3] Chen SC Tsi ZH (1998) The orchids of China. Chinese Forestry Press, Beijing, 204–206. [in Chinese]

[B4] Chen SC Wood JJ (2009) *Pholidota* Lindl. ex Hook. In: Wu ZY Raven PH Hong DY (Eds) Flora of China (Vol.25) (Orchidaceae). Science Press, Beijing and Missouri Botanical Garden Press, St. Louis, 335–339.

[B5] De Vogel EF (1988) Revision in Coelogyninae (Orchidaceae) III, The genus *Pholidota*. Orchid Monographs 3. E.J. Brill, Leiden.

[B6] Gagnepain F (1950) Orchidacées nouvelles d’Indochine. Bulletin du Muséum National d’Histoire Naturelle, série 2(22): 1–508.

[B7] Hooker WJ (1825) Exotic Flora: Containing Figures and Descriptions of New, Rare, or Otherwise Interesting Exotic Plants (Vol. II). Printed for William Blackwood, Edinburgh and T. Cadell, London.

[B8] Pearce NR Cribb PJ (2002) Flora of Bhutan (Vol. 3 part 3): The Orchids of Bhutan. Royal Botanic Garden Edinburgh & Royal Government of Bhutan, 348–354.

[B9] Pridgeon AM Cribb PJ Chase MW Rasmussen FN (2005) Genera Orchidacearum (Vol. 4): Epidendroideae (Part one). Oxford University Press, Oxford, 76–81.

[B10] Rolfe RA (1903) Orchidaceae: Epidendreae In: Formosa Hainan Corea the Luchu Archipelago and the Island of Hongkong together with their distribution and synonymy Part IV. Journal of the Linnean Society of London.Botany 36: 1–72. 10.1111/j.1095-8339.1903.tb02550.x

[B11] Schlechter FRR (1913) Orchidaceae novae et criticae. Repertorium Specierum Novarum Regni Vegetabilis. Beihefte 12: 1–107. 10.1002/fedr.19130120912

[B12] Seidenfaden G (1986) Orchid genera in Thailand XII. Thirty-three Epidendroid genera. Opera Botanica 89: 96–106.

[B13] Seidenfaden G Wood JJ (1992) The orchids of Peninsular Malaysia and Singapore. Olsen and Olsen, Fredensborg.

[B14] Thiers B (2015) Index Herbariorum: A global directory of public herbaria and associated staff. New York Botanical Garden’s Virtual Herbarium. http://sweetgum.nybg.org/ih/

